# Outer retinal tubulation in diabetic macular edema following anti-VEGF treatment

**DOI:** 10.1186/s40662-015-0018-2

**Published:** 2015-05-27

**Authors:** Ali M Al-Halafi

**Affiliations:** Department of Surgery, Ophthalmology Division, Consultant and Vitreoretinal Surgeon Security forces Hospital, PO Box 3643, Riyadh, 11481 Kingdom of Saudi Arabia

**Keywords:** Outer retinal tubulation, Diabetic macular edema, Ranibizumab, Bevacizumab

## Abstract

**Background:**

To address the presence and features of outer retinal tubulation (ORT) found in diabetic macular edema (DME) treated with anti-vascular endothelial growth factor (anti-VEGF) and to differentiate between ORT and cystoid DME, which have different plans of management.

**Methods:**

This was a retrospective review of a total of 514 patients investigated with spectral domain optical coherence tomography (OCT) in patients with diabetic macular edema treated with anti-VEGF. ORT was seen in 12 eyes of 11 patients. The morphologic characteristics of ORT and its progress over time were examined using OCT data. The retinal images were obtained by horizontal and vertical scans to analyze the possible presence of ORT and to explore their morphologic features and location in the retinal layers.

**Results:**

ORT was seen in DME treated with anti-VEGF. ORT was shown as round or ovoid hyporeflective spaces with hyperreflective borders on the B-scans, measuring 30 to 120 μm high and 30 to 1775 μm wide. The tubules generally remained stable over time. In a retinal practice specializing in advanced diabetic retinopathy clinic, this ORT was seen in 12 eyes of 11 patients during a 12-month period. ORT presented either after receiving 0.05 mL open-label intravitreal injections of 0.5 mg ranibizumab or 1.25 mg bevacizumab.

**Conclusion:**

ORT is found in DME treated with anti-VEGF that may show damage to the outer retina secondary to the severity and chronicity of the DME. ORT may be a result of underlying chronic and severe diabetic macular edema that may occur later possibly secondary to retinal layers rearrangement after several anti-VEGF injections. It is important to differentiate between ORT and cystoids DME. The presence of the ORT entity alone without the presence of DME does not require further anti-VEGF re-injections.

## Background

Vascular endothelial growth factor (VEGF) has a significant role in blood-retinal barrier breakdown, which leads to fluid leakage and the development of macular edema [1]. As VEGF intraocular levels are increased in diabetic macular edema (DME), it was hypothesized that alternative or adjunct therapies using VEGF inhibitors (anti-VEGF) could be beneficial in reversing vision loss from macular edema [2].

Optical coherence tomography (OCT) has an important clinical influence in ophthalmology, which is a promising tool for performing high-resolution cross-sectional image. The first *in vivo* tomograms of the human optic disc and macula were established in 1993 [3,4]. With the widespread adoption of spectral domain optical coherence tomography (SD-OCT) in diagnosing and following up retinal disease, outer retinal tubulation (ORT) has become a more obviously well-known presence in eyes with focal disruptions of the outer retina related to different disorders [5]. Age-related macular degeneration (AMD) and inflammatory diseases such as multifocal choroiditis or non-inflammatory diseases were observed to have ORT [5]. ORT has a single straight tubule or branching tubules to complex networks as made visible by en face SD-OCT C-scan. It is usually situated in the outer nuclear layer of the retina and as round or ovoid hyporeflective spaces with hyperreflective borders on B-scan SD-OCT sections [5].

Curcio et al. [6] were the first to identify this ORT in a histopathologic study of eyes with advanced AMD. They found that surviving photoreceptors seemed to rearrange into interconnecting tubes over disciform scars. In histopathologic and immunofluorescence studies of autopsy eyes from a patient with retinitis pigmentosa (RP), it was demonstrated that patches of remaining photoreceptors organized in rosettes, formed mainly of rods, were seen with lumens containing disorganized photoreceptor structures [6]. Histopathologically, rosettes observed in mouse models with degenerated retinas were matched to tubular elements shown in OCT findings [7]. In adult rat retinas, induced injury to photoreceptors with sparing of the retinal pigment epithelium (RPE) led to inner segment/outer segment (IS/OS) degeneration and migration of photoreceptor nuclei in a circular pattern with resultant rosette-like elements [8].

The aim of this study was to address, where possible, the presence of ORT in DME after treatment with anti-VEGF and to evaluate their impact on clinical practice.

## Methods

After approval by the research committee and human ethics committee (Kingdom of Saudi Arabia Ministry of Interior General Administration for Medical Services, Security Forces Hospital Program RN:15/148/03), a retrospective observational case study series was performed at the Security Forces Hospital, Riyadh, Saudi Arabia. The retrospective review of medical records of consecutive patients with DME treated with anti-VEGF seen at the outpatient retina clinic of January 2013 through January 2014 was included. The exclusion criteria were macular disorder associated with choroidal neovascularization (CNV), retinal dystrophies, degenerations, or inflammatory disorders other than DME. Each patient underwent ophthalmic examination (visual acuity, intraocular pressure, biomicroscopy, and dilated fundus examination) at each visit. Diagnosis of DME and management was made for each patient on the basis of fundus examination and confirmed with OCT.

A total of 514 patients with DME treated with anti-VEGF were examined with SD-OCT. ORT was identified in 12 eyes of 11 patients with an estimated incidence rate of 2.3%. The mean age ± standard deviation (SD) was 43 ± 13 years. The best-corrected visual acuities and cataracts were minimal to absent in all study subjects. All patients received 0.05 mL open-label intravitreal injections of 0.5 mg ranibizumab and bevacizumab.

The SD-OCT images of macular retinal layers were investigated in all patients with high-resolution OCT **(Optos OCT SLO,** United Kingdom) and assessed after dilation of the pupil. In each retinal OCT, high-density horizontal raster scans were used. 6-mm raster scans of the axial retinal sections was performed, which simultaneously acquired SD-OCT and near-infrared reflectance images. This enabled precise identification of corresponding sites of pathology between the different imaging modalities. Horizontal and vertical scans of the images were performed to discover the possible presence of ORT and to determine its prevalence, morphologic character, and location in the retinal structures. The occurrence of ORT was determined in each retinal exam. When serial SD-OCT studies were available, the progression of ORT was assessed over time. No clear predisposing factors were observed for ORT in DME treated with anti-VEGF such as number of injections, macular thickness, cystoid edema, patient age and duration of diabetes.

### Statistical analysis

Data were collected from the medical records of recruited subjects and stored in a spreadsheet using Microsoft Excel 2010® software. Data management and coding were then done in Excel. Data were analyzed via SPSS® version 20.0 (*IBM* Inc., Chicago, Illinois).

Descriptive analysis was primarily done, where categorical variables were presented in the form of frequencies and percentages and continuous variables in the form of mean (±SD).

Inferential analysis was done in forms of comparison of pre and post intervention means using Wilcoxon Signed Ranks test. Comparison of proportion test was conducted to assess the change in percentage of cases that needed injection (*T*-test of one sample) using StatPac (Version 15.1.0, StatPac Inc., and Bloomington, MN, USA). A confidence interval level was set to 95% where a corresponding *p*-value threshold was identified as 0.05 and any output of *p* below 0.05 would be interpreted as an indicator of statistical significance.

## Results

ORT was found in 12 eyes of 11 patients with an estimated incidence rate of 2.3%. Treatment was with Bevacizumab in 4 eyes and Ranibizumab in 9 eyes. ORT was seen in both eyes of one patient. The mean age of the patients was 50 years (range, 21–72 years); 63.6% of patients studied were male (7) and 36.4% were female (4). The median visual acuity was 20/ 30 (range, 20/20 to counting fingers). There was no significant difference between patients with ORT or without ORT in mean age or mean best-corrected visual acuity.

**Figure 1:**
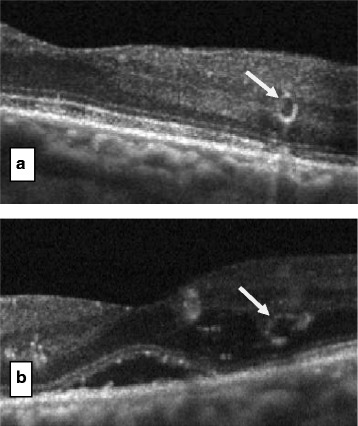
**a** & **b** Spectral-domain optical coherence tomographic scans corresponding to the solid white lines show circular outer retinal tubulation structures with hyperreflective borders.

**Figure 2:**
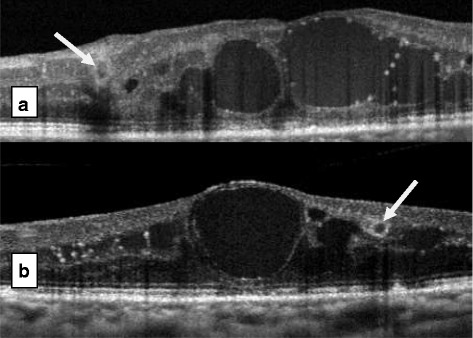
**a** & **b** Outer retinal tubulation in near proximity to sites of cystoids diabetic macular edema, demonstrating the distinguishing features of ORT (white arrows) from cystoid macular edema (black ovals).

**Figure 3:**
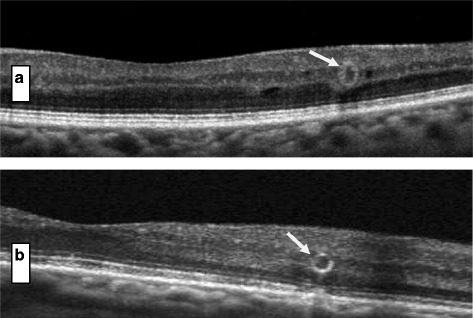
**a** & **b** Presence of ORT without thickening of the macula due to DME does not need retreatment with anti-VEGF therapy.

ORT was found in all retinal OCT as round or ovoid shapes with hyperreflective margins surrounding a central lumen with mixed reflectivity (Figure 1). In some cases, ORT was identified adjacent to the cystoid macular edema (Figure 2). ORT number in this case study ranged from 1 to 4 on any particular SD-OCT line scan. ORT vertical height ranged from 34 μm to 115 μm. ORT morphology may arise from the gradual disturbance and rearrangement of the photoreceptor layer with the IS/OS after several anti-VEGF injections secondary to severe and chronic DME. The presence of ORT alone without macular thickening does not require further anti-VEGF injections (Figure 3). It is difficult to detect ORT clinically without SD-OCT. No clear predisposing factors were observed for ORT in DME treated with anti-VEGF such as number of injections, macular thickness, cystoid edema, patient age and duration of diabetes.

## Discussion

We observed ORT in 12 eyes with DME that were treated with anti-VEGF. ORT has been detected in retinal diseases mainly associated with CNV or subretinal fibrosis, such as neovascular AMD and angioid streaks caused by pseudoxanthoma elasticum [5,6,9,10]. It has also been observed in some cases associated with geographic atrophy without CNV, such as non-neovascular AMD and gyrate atrophy [11]. 10% of patients with RP, Stargardt disease, and pattern dystrophy were reported to have ORT [12].

Despite the pathophysiology of the tubules formation being unclear, ORT appears to show a common final pathway in many retinal diseases whether they are inflammatory, degenerative or neovascular in origin. ORT has been documented in pathologies mainly involving the choroid and RPE (such as pattern dystrophy) or photoreceptors (such as RP) suggesting that ORT is a final stage in patients with chronic disorder of the photoreceptors [12]. In addition, another study has shown that the outer retinal circular or ovoid structures were reported in patients with the Bietti crystalline dystrophy [13]. The OCT findings of the Bietti crystalline dystrophy are comparable to past studies [14-16]. The SD-OCT scans distinctly showed that some hyperreflective granules were identified as crystalline deposits; despite that, there was no clear correlation between the location of ORT and crystalline deposits [17]. ORT was seen in a patient with non–AMD entities, such as chronic central serous choroidoretinopathy (CSCR), pseudoxanthoma elasticum, pattern dystrophy and multifocal choroiditis [5].

In this study, the vertical height of ORT ranged from 34 to 115 μm. Similarly, the height of ORT structures ranged from 40 to 140 μm, and the width of horizontal OCT B-scan sections ranged from 40 to 2260 μm [17]. The size of photoreceptor rosettes was reported to be around 60 μm [6,18].

Despite the pathogenesis of ORT being unclear, these differences appear to act as a late stage pathway in different retinal degenerative processes. The past reports assumed that degenerating photoreceptors may appear organized in a tubular fashion and that cells in the vicinity such as retinal pigment epithelium and glial cells may donate to the tubular structures [5]. In the OCT series, ORT was shown mainly in patients with CNV or subretinal fibrosis, and it was thought that the existence of intraretinal and subretinal exudation from CNV destroyed the photoreceptor layer, which would then activate the process of tubulation [12].

This study investigated the association between the existences of ORT in DME after treatment with anti-VEGF. The speculation is that the ORT pathogenesis in DME treated with anti-VEGF could be secondary to severe and cystoids DME as anti-VEGF therapy leads to the rearrangement of retinal layers and formation of ORT. Critical disruption of the photoreceptors and/or pigment epithelium, with or without exudative changes and scarring, seems adequate for the activity of tubulation to start [12]. This study has some limitations such as the small number DME treated with anti-VEGF patients with ORT, as well as the lack of a control. Further studies are required to deepen our understanding of ORT’s presence in patients treated for DME with anti-VEGF.

## Conclusions

ORT may be a result of underlying chronic and severe DME that may occur secondary to retinal layers rearrangement after several anti-VEGF injections. ORT is found in DME treated with anti-VEGF that may show damage to the outer retina secondary to the severity and chronicity of the DME. The differentiation between ORT and cystoids DM has a role in the treatment plan.
